# Application of statistical design for the optimization of dextranase production by a novel fungus isolated from Red Sea sponge

**DOI:** 10.1007/s13205-013-0187-4

**Published:** 2013-12-17

**Authors:** Nayera A. M. Abdelwahed, Eman Fadl Ahmed, Eman W. El-Gammal, Usama W. Hawas

**Affiliations:** 1 Pharmaceutical Industries Division, Chemistry of Natural and Microbial Products Department, National Research Centre, El-Behouth St, Dokki, Cairo, 12311 Egypt; 2 Marine Chemistry Department, Faculty of Marine Sciences, King Abdulaziz University, P. O. Box 80207, Jeddah, 21589 Kingdom of Saudi Arabia

**Keywords:** Dextranase, Fungi, Optimization, Response surface methodology

## Abstract

Marine endophytic fungi isolated from Red Sea organisms were screened for the production of dextranase enzyme. The most potent isolate was from the Red Sea sponge *Callyspongia* spp. and was selected for identification. The18S rRNA amplification for phylogenetic study revealed that the isolate was highly related to *Aspergillus flocculosus strain* NRRL 5224 by 99 %. Medium composition and culture conditions for dextranase production were optimized by response surface methodology. A significant influence of dextran, yeast extract, K_2_HPO_4_, NaNO_3_, NaCl, MgSO_4_.7H_2_O and culture requirements such as incubation time, inoculum size, medium volume and inoculum age on dextranase production was evaluated by Plackett–Burman design. The most significant factors were further optimized using Box–Behnken design. The model predicted a dextranase activity of 438.15 U/ml when dextran concentration, medium volume and incubation time were 2.1 g/l, 52.47/250 ml flask and 80.48 h, respectively. Verification of the model showed that dextranase production of 440 U/ml was observed under the optimal condition confirming the validity of the model.

## Introduction

Dextranase [(1-6)-α-d-glucan 6-glucanohydrolases] hydrolyzes the 1-6 glycosidic linkage in dextran chain (Sankpal et al. 2001). Since the first reports on *Cellvibrio fulva* dextranase in the 1940’s, more than 1,500 scientific papers and more than 100 patents have been issued on dextran-hydrolyzing enzymes found in a number of microbial groups, fungi being the most important commercial source of dextranase (Khalikova et al. 2005). Dextranase enzyme has important industrial applications: in medicine, dextranases are used for partial hydrolysis of native dextran in the preparation of blood substitutes (Molodova et al. 1980). Also, the enzyme depolymerizes various troublesome microbial dextran deposits in teeth and prevents tooth decay. It is also used for preparing low molecular weight dextran and cytotoxic dextran conjugate and dextranase is showed as an enhancer of antibiotic activity in endocarditis (Khalikova et al. 2005; Eggleston and Monge 2004; Marotta et al. 2002; Mghir et al. 1994). Since polysaccharides interfere in sugar manufacturing process in addition to loss of sucrose resulting in heavy loss to sugar factories (Priyanka and Santosh 2011), thus for improving factory performance, removal of dextran is essential. The use of dextranase in sugar mills not only improves the factory performance, but also improves the sugar quality (Fulcher and Inkerman 1974). The excessive elongation of the crystal and viscosity of syrups and molasses may be reduced by the enzymatic decomposition of dextran (Hidi 1975). In the case of metabolite production by sponge-associated microorganisms, previous research has mainly focused on marine enzymes such as lipase, chitinase and alpha-amylase (Zhang et al. 2009; Han et al. 2008; Kar et al. 2009), but their production by microbial cultivation has rarely been reported (Chu et al. 2012). Besides using a productive strain, optimized culture medium composition and fermentation conditions play a significant role in the improvement of dextranase production. Among the techniques used in optimizing the culture medium, experimental statistical techniques such as Plackett–Burman design (PB) (Plackett and Burman 1946) and response surface methodology (RSM) have proved to be the most effective methods in optimizing the medium composition for enzyme production, eliminating the limitations of single-factor optimization process (Salihu et al. 2011). In this study, statistical optimization of medium constituents and cultural conditions was employed to enhance dextranase production. In the first step, a Plackett–Burman design was used to determine the likely effects of ten possible medium variables on dextranase production. Subsequently, the most significant factors affecting dextranase production were optimized using Box–Behnken design and response-surface analyses. To the best of our knowledge, there is not enough information concerning optimization of nutritional conditions for dextranase production by marine fungi using statistical experimental designs.

## Materials and methods

### Sponge, fungal strain and media

The Red Sea sponge *Callyspongia* spp. (Phylum Porifera, class Demospongiae, order Haplosclerida family Callyspongiidae) was collected from 1.5 to 20 m depth off the coast of Egyptian Red Sea. The collection sites included the protected area of Rass Mohamed, Nabq (Aqaba Gulf) and Suez Gulf (from 60 km south Suez to south Hurgada). In the laboratory, the specimens were washed by water and processed immediately. Coral Reef Ecology and Biology group, National Institute of Oceanography and Fisheries, Suez, Egypt, identified the collected marine samples. Host surface was treated with 70 % ethanol, rinsed with sterile distilled water to remove the ethyl alcohol. Small pieces of inner tissue were rinsed with sterile sea water aseptically, then the inner tissue was cut into small cubes. A total of 3–5 cubes of each sample were placed after removing any excess moisture on different isolation media containing penicillin benzyl sodium salt to avoid any bacterial growth. After 7 days of incubation, hyphae tips of the fungi were removed and transferred to potato dextrose agar plates (PDA) that contains the following components (g/l): potato peeled and diced into small pieces 200, glucose 10, agar 15 and the pH 7.5 adjusted in 40 % sea water. After 6–7 days, velvety colonies were observed.

Screening of fungi for dextranase production was carried out using blue-dextran as described by Barrett and Curtiss (1986). The isolate giving the highest ratio of zone of clearance to colony diameter was chosen for further studies. Dextranase production was carried out in the medium with contains (g/l): dextran with average molecular weight (240.000) 1, yeast extract 2.0, K_2_HPO_4_ 0.5, NaNO_3_ 2.0, NaCl 0.1, MgSO_4_.7H_2_O 0.5 for optimization studies (Pleszczynska et al. 1997) at 30 °C in a rotatory shaking (200 rpm). The enzyme dextranase was estimated in the broth medium after the removal of fungal mycelium.

### Molecular identification

#### Preparation of the fungal cultures

Spores of the isolated marine strain were inoculated in 20 ml of 20 % potato dextrose Agar (PDA) media and incubated in 28 ± 1 °C for 3 days. The cultures were filtered, the mats were collected and washed by distilled water prior to genomic DNA extraction. DNA was isolated from the mycelium using the DNA easy Mini Kit (Qiagen, Tokyo, Japan) according to the manufacturer’s instructions. The DNA solution was used as the template for PCR. The oligonucleotide primers used for 18S rDNA partial sequence specific PCR were ITS1: 5′-TCCGTAGGTGAACCTGCGG-3′ and ITS4: 5′-TCCTCCGCTTCTTGATATGC-3′. The reaction mixture consisted of 1 μl of template solution, 2.5 μl of each primer (2 pmol), Go Taq flexi (2.5 U/μl, Promega) as taq polymerase, 5 μl of dNTPs (10 mM) and 5 μl of 10× reaction buffer, sterilized distilled water was added to increase the volume to 50 μl. The PCR cycling conditions were one cycle of 94 °C for 3 min, 30 cycles of 94 °C for 30 s, 50 °C for 2 s, 74 °C for 30 s and final extension step at 20 °C for 3 min. The PCR products were purified by GENECLEAN Kit (Q-BIOgene) according to the manufacture’s instruction. Purified PCR products were used in sequencing reactions with the same primers in both directions using a BigDye Terminator v3.0 Cycle Sequencing Kit (Applied Biosystems). Sequencing was performed on ABI3730XLS (Applied Biosystems). After construction of the retrieved sequence, the whole sequence was used for searching of compatible sequences from database (http://blast.ncbi.nlm.nih.gov/Blast.cgi). The phylogenetic profile of our samples was constructed using multiple sequence alignment software http://blast.ncbi.nlm.nih.gov/Blast.cgi?CMD=Web&PAGE_TYPE=BlastNews declaring the molecular identities with the closely related isolates in the database (Zheng et al. 2000).

#### Assay of dextranase activity

Dextranase activity was assayed by the method of Janson and Porath (1966). Reaction mixture containing 2 ml of 2.5 % dextran in acetate buffer (0.1 M, pH 5.6) and 1 ml of enzyme in a total volume of 3 ml was incubated at 40 °C for 20 min. Reaction was stopped by adding 3 ml of DNS (dinitrosalicylic acid) reagent and absorbance was measured at 550 nm in UV/Vis spectrophotometer. One dextranase unit (U) is defined as the amount of enzyme which releases 1 μmol of reducing end groups of glucose per minute.

### Optimization of process parameters

#### Identification of suitable variables using Plackett–Burman (PB) design

The Plackett–Burman was employed for screening the most significant fermentation parameters affecting dextranase enzyme production by fungal strain isolate (Sastry and Khan 1998). The variables chosen for the present study including medium components such as dextran, yeast extract, K_2_HPO_4_, NaNO_3_, NaCl and MgSO_4_·7H_2_O also operating conditions such as incubation periods, inoculum size, medium volume and inoculum age; ten assigned variables in PB design of 12 experiments. Each independent variable was tested at two levels, high and low, which are denoted by (+) and (−), respectively. The experimental design with the name, symbol code, and actual level of the variables is shown in Table 1, whereas Table 2 shows the details of the design. PBD is based on the first order polynomial model:

**Table 1:** Experimental definition for the Plackett–Burman design

Symbol code	Factors	Experimental values	
		Low level (−1)	High level (+1)
*X*_1_ (g/l)	Dextran	0.5	1.5
*X*_2_ (g/l)	Yeast extract	1	2
*X*_3_ (g/l)	K_2_HPO_4_	0.5	1
*X*_4_ (g/l)	NaNO_3_	1	2
*X*_5_ (g/l)	NaCl	0.5	1
*X*_6_ (g/l)	MgSO_4_·7H_2_O	0.5	1
*X*_7_ (days)	Incubation time	5	7
*X*_8_ (ml)	Inoculum size	1	2
*X*_9_ (ml/250 ml flask)	Medium volume	25	50
*X*_10_ (h)	Inoculum age	48	72

**Table 2:** Two-level factorial design of variables (in coded levels) with titer as response values

Run	*X*_1_	*X*_2_	*X*_3_	*X*_4_	*X*_5_	*X*_6_	*X*_7_	*X*_8_	*X*_9_	*X*_10_	Dextranase activity (U/ml)
1	1	−1	1	1	1	−1	1	−1	−1	−1	358.5
2	1	−1	−1	1	1	1	−1	1	1	−1	327.0
3	−1	−1	−1	−1	1	1	1	−1	1	1	351.0
4	1	1	1	−1	−1	1	1	1	−1	−1	321.9
5	1	−1	1	−1	−1	−1	−1	1	1	1	420.6
6	1	1	−1	1	−1	−1	−1	−1	1	1	499.5
7	−1	1	1	−1	1	−1	1	1	1	−1	265.2
8	−1	−1	1	1	−1	1	1	1	−1	1	274.8
9	−1	1	−1	1	1	−1	−1	1	−1	1	212.1
10	1	1	1	−1	1	1	−1	−1	−1	1	333.9
11	−1	1	1	1	−1	1	−1	−1	1	−1	349.5
12	−1	−1	−1	−1	−1	−1	−1	−1	−1	−1	321.9

Variables coded are same as given in Table 1

1 $$Y\;=\beta_{o}+\sum \beta_{i}X_{i}$$

where *Y* is the response (dextranase enzyme production), *β*_*o*_ is the model intercept*, β*_*i*_ is the linear coefficient and *X*_*i*_ is the level of the independent variable. All the experiments were carried out in duplicates and the averages activity is reported as the final response in Table 2.

#### Box–Behnken design

From the regression analysis of the variables, the most significant factors for dextranase enzyme production were further optimized by the Box–Behnken statistical design (Box and Behnken 1960). They were further analyzed at three levels of concentration to find out the most optimal values for producing highly active dextranase enzyme. The three levels were coded as −1, 0 and +1 representing low, middle and high concentrations, respectively, as shown in Table 3. According to the design, 15 combinations were tested (Table 4) and their observations were fitted to the following second order equation as represented in Eq. 2 as follows,

**Table 3:** Coded and actual values of the culture conditions tested in Box–Behnken design

Symbol code	Factors	Actual levels of coded factors		
		+1	0	−1
*X*_1_ (g/l)	Dextran	2.5	2.0	1.5
*X*_9_ (ml/250 ml flask)	Medium volume	65	50	25
*X*_10_ (h)	Inoculum age	96	72	48

**Table 4:** Box–Behnken of three variables in coded along with titer as response values

Run	*X*_1_	*X*_9_	*X*_10_	Dextranase activity (U/ml)
1	−1	−1	0	326.70
2	1	−1	0	348.06
3	−1	1	0	357.70
4	1	1	0	414.90
5	−1	0	−1	271.80
6	1	0	−1	362.40
7	−1	0	1	393.60
8	1	0	1	398.40
9	0	−1	−1	257.70
10	0	1	−1	403.20
11	0	−1	1	395.66
12	0	1	1	403.20
13	0	0	0	429.00
14	0	0	0	429.00
15	0	0	0	429.00

2 $$Y\;=\;\beta_{o}+\;\beta_{1}X_{1}+\;\beta_{2}X_{2}+\;\beta_{3}X_{3}+\beta_{11}X_{1}^{2}+\beta_{22}X_{2}^{2}+\beta_{33}X_{3}^{2}+\beta_{12}X_{1}X_{2}+\beta_{13}X_{1}X_{3}+\beta_{23}X_{2}X_{3}$$

where *Y* is the measured response (dextranase enzyme production), *X*_1_, *X*_2_ and *X*_3_ are independent variables, which influence the response variable *Y*, *β*_1_, *β*_2_, *β*_3_ are linear coefficients, *β*_12_, *β*_22_ and *β*_33_ are cross-product coefficients and *β*_11_, *β*_22_ and *β*_33_ are quadratic coefficients. The quality of the fit of the polynomial model equation is expressed by the coefficient of determination *R*^2^.

#### Data analysis and optimization

The data of the enzyme activity of each trial was subjected to analysis, using statistical tool Minitab 16 software for Plackett–Burman and the Box–Behnken experiment. Statistical analysis of the model was performed through the analysis of variance (ANOVA) to evaluate the statistical significance of the model. The models of each response were expressed in terms of uncoded variables. The quality of the polynomial model equation was judged statistically by the coefficient of determination *R*^2^, adjusted *R*^2^, and predicted *R*^2^. The determination coefficient (*R*^2^) is a measure of how well the regression equation fits the sample data. Adjusted *R*^2^ is a modification of *R*^2^ that adjusts for the number of explanatory terms in a model. A predicted *R*^2^ is used to measure the amount of variation in new data (i.e., other levels among the tested maximal value and minimal value of the factor) explained by the model. Statistical significance was determined by an *F* test at 5 % level. The significance of the regression coefficients was tested by a *t* test. STATISTICA sigma software (Version 8.0, StatSoft Inc., USA) was used to plot Pareto chart of standardized effects, the three-dimensional surface plots and contour plots, in order to illustrate the relationship between the response and the experimental levels of each of the variables utilized in this study.

## Results and discussion

### Identification of active marine endophyte

The aim of this study was to find strains of marine fungi for the production of dextranase with potential industrial applications. The identification of fungi is mainly done by recognizing the morphological features of genera and species by macroscopic and microscopic examination. Among molecular techniques, PCR-specific amplification is a rapid method used in the direct detection of DNAs and RNAs of microorganisms from clinical and environmental samples to accurately and quantitatively ascribe microorganism compositions (Zhihong et al. 2003). The fungal strain isolated from Red Sea sponge *Callyspongia* spp. exhibited dextranase activity on dextran medium. It gave a zone of clearance to colony diameter ratio of 2.0 cm which was higher than the clearance displayed by other strains obtained from the region and was chosen for molecular identification technique. According to sequencing similarities and multiple alignment, the fungus was found to be in a close relation to *Aspergillus flocculosus* strain NRRL 5224 (ac: EU021616.1) with a 99 % identity (Table 5); accordingly, the strain is hereafter referred to as *Aspergillus flocculosus* EU NRC. Since the organism appeared promising, experiments were devised to optimize the cultural conditions. It was reported before that, fungi and bacteria were identified as the main enzymatic sources capable of hydrolyzing dextrans. In the early 1950s Japanese researchers identified *Penicillium lilacinum* and *Penicillium funiculosum* fungi that produced dextranase in the presence of dextrans and later in the 1960s others strains from *Chaetomium gracile* and *Gibellela funiculosum* fungi. After an extensive search that continued in Japan during the 1970s, the *Aspergillus carneus* fungus strain that accumulates the enzyme when cultured in dextrans and another from the *Penicillium luteum* were identified (Novo 1977). The dextranase from *P*. *lilacinum* showed maximum activity in the pH range of 5.0–5.5 and between 53 and 60 °C (Fukumoto et al. 1971). Similar observations were reported for the production of dextranase by various fungi (Hattori and Ishibashi 1981; Shukla and Madhu 1989; Madhu 1984).

**Table 5:** Distribution of the first ten blast hits on the query sequence

Organisms description	GenBank accession	Identities		
		Match	Total	Pct. (%)
*Aspergillus flocculosus* strain NRRL 5224	EU021616.1	587	589	99
*Aspergillus* sp. r089	HQ649845.1	582	587	99
*Aspergillus ochraceopetaliformis* strain RKI08-134	FJ797698.1	583	589	99
*Aspergillus ochraceopetaliformis* isolate NRRL 35668	EF661432.1	575	577	99
*Aspergillus ochraceopetaliformis* isolate NRRL 35055	EF661431.1	575	577	99
*Aspergillus ochraceopetaliformis* isolate NRRL 4752	EF661429.1	575	577	99
*Aspergillus* sp. F5	FJ214372.1	581	588	99
*Aspergillus* sp. r192	HQ649847.1	576	581	99
*Aspergillus* sp. OY10607	FJ571434.1	567	568	99
*Aspergillus ochraceopetaliformis* strain SCSGAF0071	JN851013.1	563	568	99

### Screening of important variables for dextranase production using Plackett–Burman design

The influence of dextran, yeast extract, K_2_HPO_4_, NaNO_3_, NaCl, MgSO_4_·7H_2_O concentrations as well as incubation time, inoculum size, medium volume and inoculum age on dextranase enzyme production were investigated with the help of PBD. According to Davis et al. (1974) corn steep liquor and autolyzed yeast extract were commonly used for the dextranase enzyme production. The data in Table 2 indicated that there was a wide variation from 212 to 499.5 U/ml of dextranase enzyme in the 12 runs which reflects the variations caused due to the presence of different factors influencing the activity at low and high levels considered in various combinations.

Regression analysis and the analysis of variance (ANOVA) showed probability values <0.05 (*p* < 0.05) indicated significance of the model term, whereas *p* values >0.1 (*p* > 0.1) indicated model terms were not significant as shown in Tables 6 and 7. The *p* value is the probability that the magnitude of a contrast coefficient is due to random process variability and serves as a tool for checking the significance of each of the coefficients, low *p* value indicates a real or significant effect (Levine 2005).On the same basis, dextran (*t* = 55.97, *p* = 0.011), medium volume (*t* = 44.79, *p* = 0.014) and inoculum age (*t* = 17, *p* = 0.037) with higher *t* value and lower *p* values were considered as the most significant components (Table 6). When the sign of the concentration effect of the tested variable is positive, the influence of the variable upon dextranase enzyme production is greater at a high concentration and when negative, the influence of the variable is greater at a low concentration. The effect of variables *X*_2_ (yeast extract), *X*_5_ (NaCl), *X*_6_ (MgSO_4_·7H_2_O) and *X*_8_ (inoculum size) are −23.9, −227, −79.8 and −130.9, respectively, i.e., the influence of these four variables is greater at a low concentration. The goodness of the fit of the model can be checked by the ‘determination coefficient’ *R*^2^, the value of *R*^2^ and adjusted *R*^2^ are 99.9 and 99.8 %, respectively, which shows a high correlation between the observed values and the predicted values. This means that regression model provides an excellent explanation of the relationship between the independent variables (factors) and the response (dextranase enzyme production). On application of ANOVA, it was found that the first order model for dextranase enzyme production was fitted to the results obtained from the 12 experiments as the equation:

**Table 6:** Estimated regression coefficients for dextranase enzyme production

Code	Variable	Effect	*t* test	*F* ratio	*B* coefficient	*p* value
*X*_1_	Dextran (g/l)	162.3	55.97	3,132.14	81.1500	0.011*
*X*_2_	Yeast extract (g/l)	−23.9	−8.24	67.92	−11.9500	0.077
*X*_3_	K_2_HPO_4_ (g/l)	−10.300	−3.55	12.61	−10.3	0.175
*X*_4_	NaNO_3_ (g/l)	2.3	0.79	0.63	1.15000	0.573
*X*_5_	NaCl (g/l)	−227.0	−39.14	1,531.78	−113.500	0.016*
*X*_6_	MgSO_4_·7H_2_O (g/l)	−79.8	−13.76	189.30	−39.9	0.046
*X*_7_	Incubation time (days)	17.6	12.17	148.17	8.82500	0.052
*X*_8_	Inoculum size (ml)	−130.9	−45.14	2,037.43	−65.4500	0.014*
*X*_9_	Medium volume (ml/250 mlflask)	5.2	44.79	2,006.42	2.59800	0.014*
*X*_10_	Inoculum age (h)	2.1	17.00	289.00	1.02708	0.037*

* Identifies variables with a significant effect on the response (*p* value <0.05)

$$Y\;=\;280\;+\;81.15\;X_{1}-\;11.95\;X_{2}-\;10.3\;X_{3}+\;1.15\;X_{4}-\;113\;X_{5}-\;39.9\;X_{6}+\;8.83\;X_{7}-\;65.5\;X_{8}+\;2.60\;X_{9}+\;1.03\;X_{10}$$

where, *Y* predicted response and *X*_1_, *X*_2_, *X*_3_, *X*_4_, *X*_5_, *X*_6_, *X*_7_, *X*_8_, *X*_9_ and *X*_10_ are the coded values of dextran, yeast extract, K_2_HPO_4_, NaNO_3_, NaCl, MgSO_4_·7H_2_O, incubation time, inoculum size, medium volume and inoculum age, respectively.

**Fig. 1:**
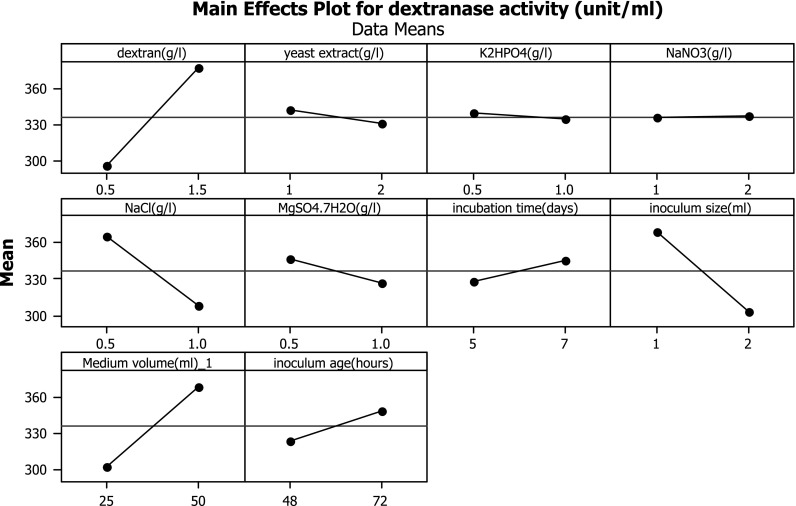
Figure shows the main effect plots for the system, which show how each factor affects the response characteristic

**Fig. 2:**
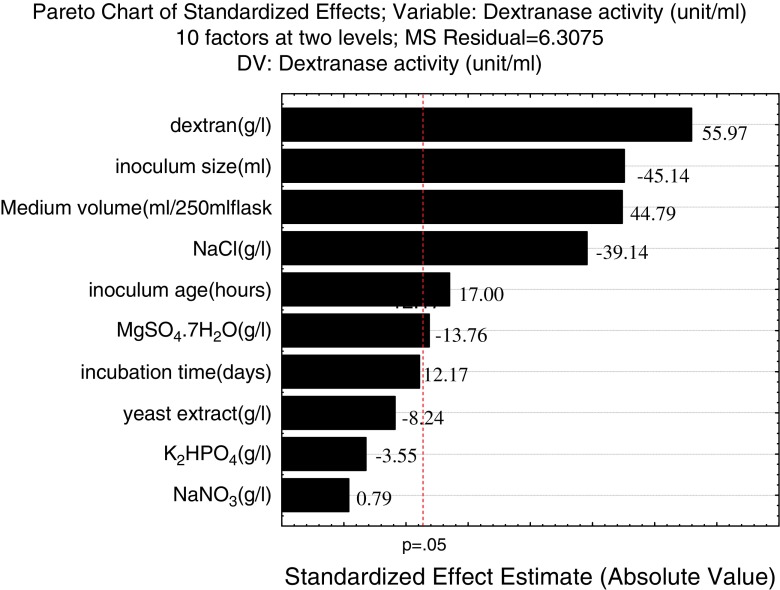
Pareto chart of standardized effects on the dextranase enzyme production. The above results indicated that the Plackett–Burman design is a powerful tool for identifying factors, which had significant influence on dextranase activity. The exact optimal values of the most significant factors (dextran, medium volume and inoculum age) can be determined by the subsequent Box–Behnken experiment. Optimization of significant culture parameters with RSM based on the Box–Behnken design. The culture variables found to significantly affect dextranase enzyme production were tested at values given in Table 6 for the Box–Behnken design to optimize the magnitude of those variables

The main effect is present when different levels of a factor affect the characteristic differently. Minitab statistical software generates the main effect plot by plotting the characteristic average for each factor level (Fig. 1). These averages are the same as those shown in Table 6. A line connects the points for each factor. When the line is horizontal (parallel to the *x*-axis), main effect is not present. Different levels of the factor affect the characteristic differently. The greater the difference in the vertical position of the plotted points (the more the line is not parallel to the *x*-axis), the greater the magnitude of the main effect. Analysis of the measured response variables enabled to obtain standardized Pareto charts and response surface plots. A standardized Pareto chart consists of bars with a length proportional to the absolute value of the estimated effects, divided by the standard error. The bars are displayed in order of the size of the effects, with the largest effects on top. A high *t* test value and a low probability indicated a high significance (Niladevi et al. 2009). The chart includes a vertical line at the critical *t* value for an alpha of 0.05. Bars are displayed in order of the size of the effects, and the standardized effect of each term was shown on the top of its corresponding bar (Fig. 2).

**Table 7:** Analysis of variance (ANOVA) for the quadratic model

Source	*df*	SS	MS	*F*	*p*
Regression	10	59,387.7	5,938.8	941.54	0.025
Residual error	1	6.3	6.3		

*R*^2^ = 99.99 %, *R*^2^ (pred) = 98.47 %, *R*^2^ (adj) = 99.88 %

*SS* sum of squares, *df* degrees of freedom, *MS* mean square

Results showed that, variations of dextranase production in the 15 trials ranged from 257.70 to 429 U/ml (Table 4).

Regression analysis in Table 8 of the experimental data shows that dextran, medium volume and inoculum age had positive linear effects on enzyme synthesis (*p* < 0.05). Probability (*p*) values were used as a tool to check the significance of each of the coefficients. The smaller the magnitude of *p* value, the more significant was the correlation with the corresponding coefficient (Sreekumar and Krishnan 2010). Among the three factors tested, inoculum age had the highest impact on dextranase enzyme activity as given by the highest linear coefficient (732.90), followed by inoculum age (17.03) and medium volume (9.83). These factors also showed significant negative quadratic effects on enzyme production indicating that dextranase enzyme activity increased as the level of these factors decreased and decreased as the level of these parameters increased above certain values. Interaction between these parameters was also significant. The interactions between dextran concentration–inoculum age and medium volume–inoculum age were significant as shown by low *p* values (*p* < 0.05) for interactive terms. But the interaction between dextran concentration–medium volume was found to be insignificant as given by *p* value above 0.05. Hence this term was excluded from the quadratic polynomial Eq. 2 used for this model.

**Table 8:** Estimated regression coefficients of Box–Behnken design for dextranase enzyme production

Term		Coefficients	SE coefficients	*t* value	*p* value
Constant		−1,282.69	161.252	−7.955	0.001*
Dextran	*X*_1_	732.90	112.166	6.534	0.001*
Medium volume	*X*_9_	9.83	2.143	4.586	0.006*
Inoculum age	*X*_10_	17.03	1.987	8.572	0.000*
Dextran × dextran	*X*_1_ × *X*_1_	−151.10	25.430	−5.942	0.002*
Medium volume × medium volume	*X*_9_ × *X*_9_	−0.06	0.017	−3.341	0.021
Inoculum age × inoculum age	*X*_10_ × *X*_10_	−0.06	0.011	−5.454	0.003*
Dextran × medium volume	*X*_1_ × *X*_9_	0.92	0.601	1.529	0.187
Dextran × inoculum age	*X*_1_ × *X*_10_	−1.79	0.509	−3.512	0.017*
Medium volume × inoculum age	*X*_9_ × *X*_10_	−0.07	0.013	−5.460	0.003*

*R*^2^ = 98.19 %, *R*^2^ (pred) = 68.97 %, *R*^2^ (adj) = 94.93 %

*SE* standard error, *t* Student’s test, *p* corresponding level of significance

* Significant

$$Y=-1,282.69+732.90\;X_{1}+9.83\;X_{9}+17.03\;X_{10}-151.10\;X_{1}X_{1}-0.06\;X_{9}X_{9}-0.06\;X_{10}X_{10}-1.79\;X_{1}X_{10}-0.07\;X_{9}X_{10}$$

where *Y* is the response (dextranase enzyme activity) and *X*_1_, *X*_9_ and *X*_10_, are the coded values of the independent variables.

The adequacy of the model was checked using analysis of variance (ANOVA) and the results were presented in Table 9. The correlation coefficient (*R*^2^ = 98.19 % and adjusted coefficient *R*^2^ (adjusted) = 94.93 % were also high, which indicates a high significance of the experiments (Akhnazarova and Kafarov 1982).

**Table 9:** ANOVA for response surface quadratic model

Source	Degrees of freedom	Sum of square	Adjusted sum of square	Mean of square	*F* value	*p* value
Regression	9	40,429.4	40,429.4	4,492.2	30.10	0.001
Linear	3	23,800.7	15,210.1	5,070.0	33.97	0.001
Square	3	9,989.6	9,989.6	3,329.9	22.31	0.003
Interaction	3	6,639.1	6,639.1	2,213.0	14.83	0.006
Residual error	5	746.2	746.2	149.2		
Lack-of-fit	3	746.2	746.2	248.7		
Pure error	2	0.0	0.0	0.0		
Total	14	41,175.6				

*R*^2^ = 98.87 %, *R*^2^ (pred) = 81.91 %, *R*^2^(adj) = 96.83 %

*F* Fishers’s function, *p* corresponding level of significance

**Fig. 3:**
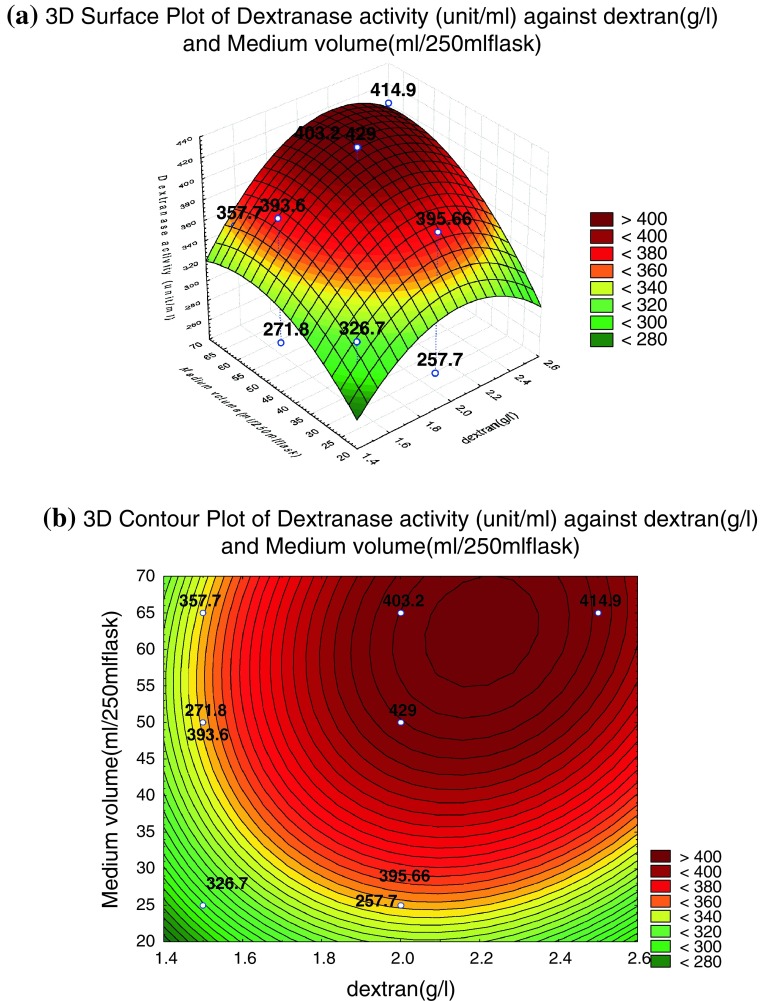
**a** Response surface and **b** contour plots for the interaction of dextran concentration and medium volume at inoculum age 72 h on dextranase enzyme activity. The *values in the figure* indicated the level of dextranase enzyme activity (U/ml)

The ANOVA of the regression model demonstrates that the model is highly significant. This is evident from the calculated *F* value (Thompson et al. 1994; Khuri and Cornell 1987) where (*F* model = 30.10) and probability value (*p* = 0.001). It is evident that the linear (*p* = 0.001) quadratic effect (*p* = 0.003) and interaction effect (*p* = 0.006) of the variables had greater influence on dextranase enzyme production. The model determination coefficient *R*^2^ was reasonable and in agreement with the experimental results, indicating that 98.87 % of the variability could be revealed by the model (Table 9). Accordingly, three-dimensional graphs were generated for the pair-wise combination of the three factors, while keeping the third one at its middle point levels. From the middle point of the bump of the 3D plot, the optimal conditions of the production medium was identified. Figure 3a represent three-dimensional curve of the calculated response surface from the interaction effect of dextran concentration and medium volume on dextranase enzyme production, whereas inoculum age was kept at its middle level, that is, 72 h. The response surface plot indicated that dextranase enzyme production increased with the increase in both dextran concentration and medium volume. With further increase in dextran concentration and medium volume, the yield slightly decreased this is may be due to excess accumulation of dextran in medium which increases the viscosity and ultimately hinders the fungal growth, resulting in decreased production of dextranase.

**Fig. 4:**
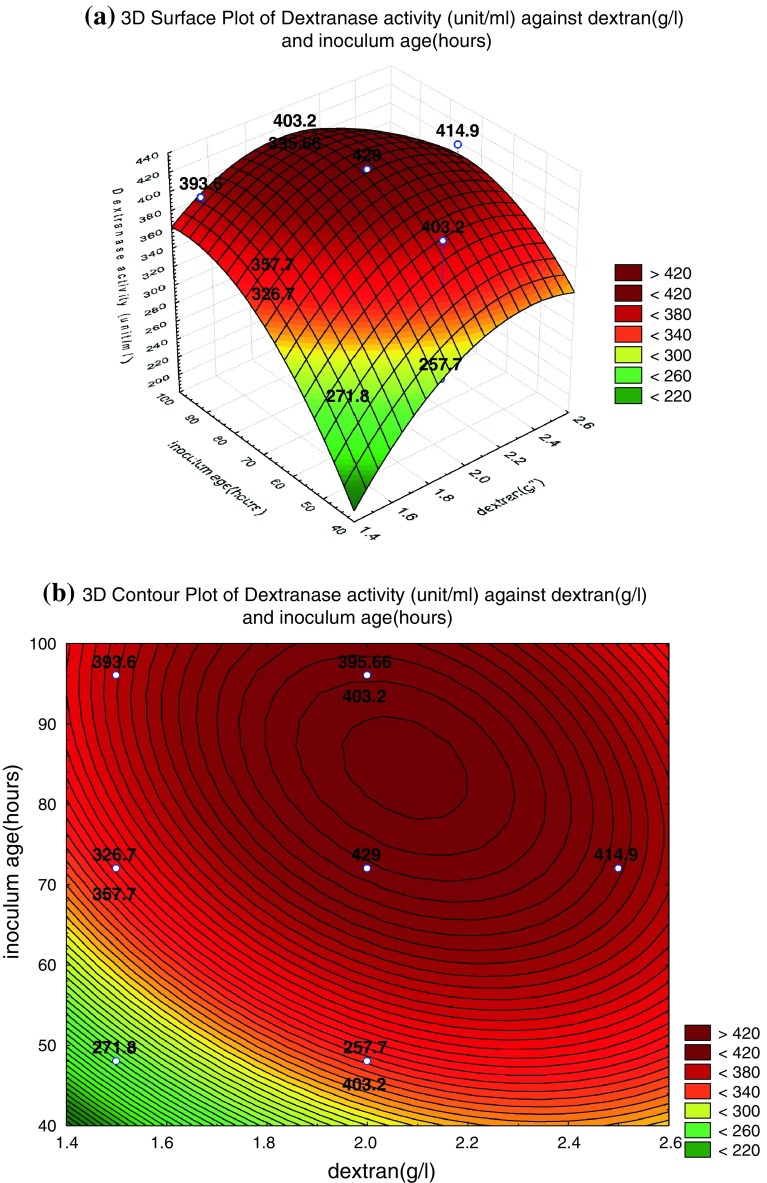
**a** Response surface and **b** contour plots for the interaction of dextran concentration and inoculum age at medium volume 50/250 ml flask on dextranase enzyme activity. The *values in the figure* indicated the level of dextranase enzyme activity (U/ml)

Figure 4a shows the interaction effect of dextran concentration and inoculum age on dextranase enzyme production, whereas medium volume was kept at its middle level, 50/250 ml flask. The dextranase enzyme production increased with the increase of medium volume with further increase in inoculum age, the yield slightly decreased which may be due to the occurrence of microbial death phase.

**Fig. 5:**
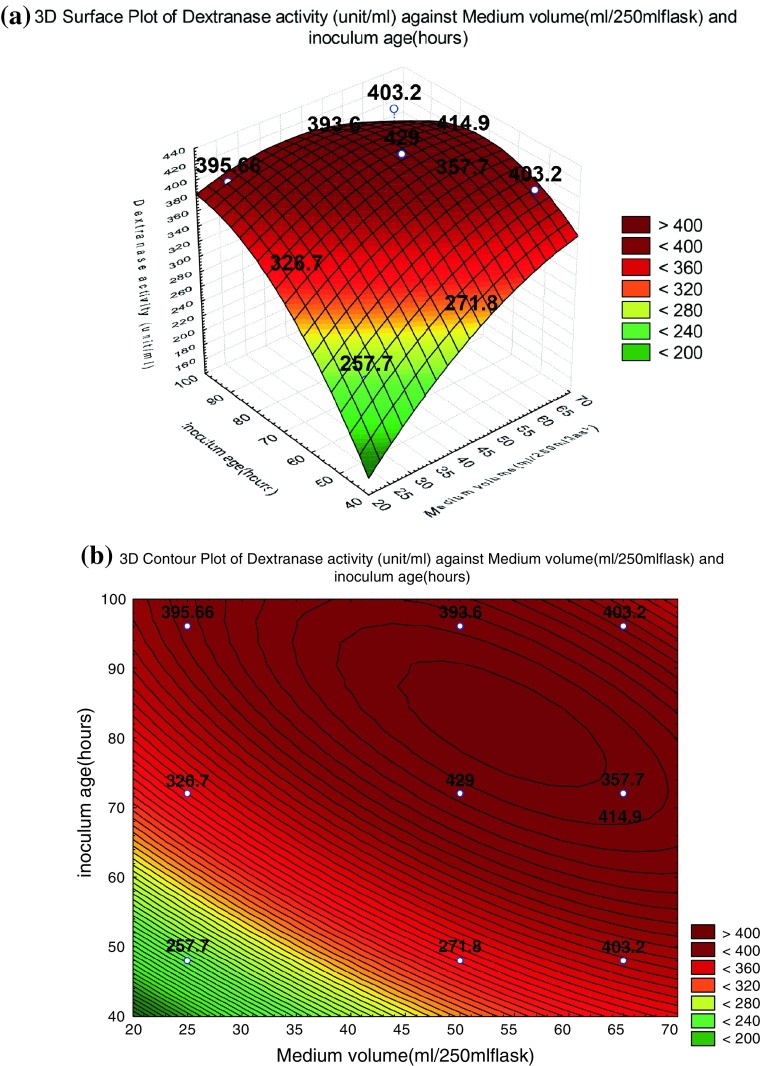
**a** Response surface and **b** contour plots for the interaction of medium volume (ml/250 ml flask) and inoculum age (h) at dextran concentration 2 g/l on dextranase enzyme activity. The *values in the figure* indicated the level of dextranase enzyme activity (U/ml)

Figure 5a represents the interactive effect of medium volume and inoculum age on dextranase enzyme production, whereas dextran concentration was kept at its middle level, 2 g/l. The production increased with the increase of medium volume and inoculum age, the yield slightly decreased with further increase in medium volume and inoculum age which may be due to the occurrence of microbial death phase.

The yield values for different concentrations of the variables can also be predicted from the respective contour plots (Box et al. 1978; Box and Wilson 1951; Khuri and Cornell 1987). The maximum predicted yield is indicated by the surface confined in the smallest ellipse in the contour diagram. The contour plots for each of the responses generated are shown in Figs. 3, 4, 5b. All the contour plots are elliptical indicating that there is a perfect interaction between the independent variables (Muralidhar et al. 2001). Higher production was recorded in Fig. 3b when dextran concentration is in the range of 2.1–2.3 g/l and medium volume is in the range of 52–70 (ml/250 ml flask). Similar pattern of highest enzymatic production was also recoded from Fig. 4b when dextran concentration is in the range of 2–2.2 g/l and the inoculum age is in the range of 80–90 h, whereas Fig. 5b shows medium volume of 45–65 (ml/250 ml flask) and inoculum age is in the range of 70–90 h.

**Fig. 6:**
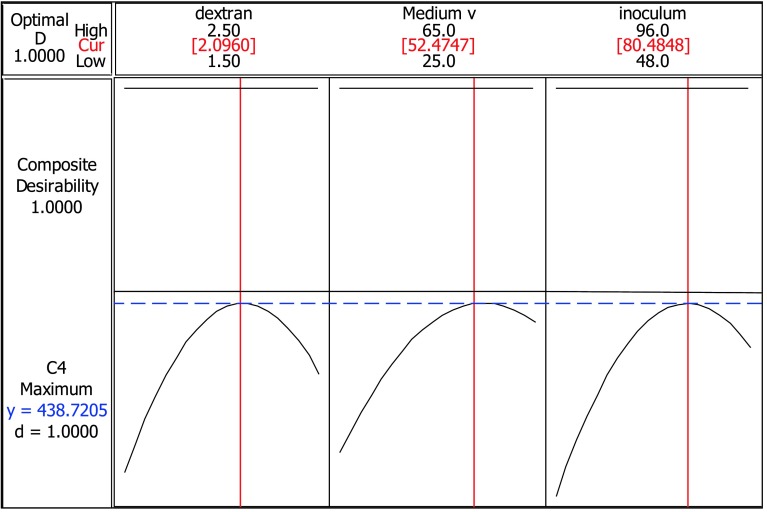
Recommended input variables to achieve optimal response

Eventually, after making the regression model, a numerical optimization method by desirability function was implied to optimize the response. The graph in Fig. 6 indicates how individual factors in each column influence the response while the other factors are held constant. The values between high and low values optimal parametric setting were recommended by the minitab 16 software to obtain the most suitable responses. *D* is the composite desirability and *d* is the individual desirability. The maximum values for *D* and *d* are 1.0000 (Myers and Montgomery 1995). Figure 6 shows values for *D* and *d* in optimal conditions as 1.0000, confirming that the model proposed is suitable.

In the recommended optimal model (see Fig. 6), parametric settings of dextran concentration of 2.09 g/l, medium volume of 52.5/250 ml flask and inoculum age of 80.5 h, were set. The response for this set of values for dextranase enzyme production with desirability of 1.0000 was 438.72 U/ml.

Therefore, the predicted optimum condition was verified experimentally and compared with the predicted data. The measured dextranase activity was 440 U/ml higher than many fungi which synthesizes dextranase including *Penicillium funiculosum*, *P. notatum*, *Fusarium moniliforme*, *Aspergillus carneus* (Abdel-Naby et al. 1999; Fukumoto et al. 1971; Pleszczynska et al. 1997; Hiraoka et al. 1972; Simonson et al. 1975). The verification revealed a high degree of accuracy of the model of more than 96.7 %, indicating the model validation under the tested conditions. Therefore, this *Aspergillus flocculosus* EU NRC strain is of interest for further studies.

## Conclusion

The reported fungus could be used for industrial production of dextranase as it is a non-pathogenic strain which grows at mild conditions and produces extracellular, highly substrate-specific dextranase for the removal of dextran contamination. In this work, Plackett–Burman design was used to determine the relative importance of medium conditions for dextranase production. Among the variables, dextran, medium volume and inoculum age were found to be the most significant variables. From further optimization studies, the optimized values of the variables for maximum dextranase production were as follows: dextran concentration of 2.09 g/l, medium volume of 52.5/250 ml flask and inoculum age of 80.5 h. The maximum production of dextranase was obtained under the optimized media 440 (U/ml). The results show a close concordance between the predicted and the experimental run. These results may provide an important basis for industrial applications.
